# Supplementary material for: Content-rich biological network constructed by mining PubMed abstracts
Source: BMC Bioinformatics. 2004 Oct 8;5:147. doi: 10.1186/1471-2105-5-147 (PMC528731; doi:10.1186/1471-2105-5-147)
Supplement: Additional File 2 — The original results of the above study (non-essential files are deleted to keep the file size under the limit set by BMC bioinformatics). [file 1471-2105-5-147-S2.bz2 › chilibotAdditionalFile2/dip30/25ID8999548E88/html/CASP1_IL1B.html]

 


 **CASP1** and **IL1B** 
  
Found 435 abstracts in PubMed, retrieved 30.  
 

 What does Google say? 
 PDF only 
| .edu only 

---

**Interactive relationship** (e.g. stimulation, inhibition, etc)

**Stimulatory relationship**- Furthermore, treatment with the IL 1 beta  [ **IL1B** ]  converting enzyme  [ **CASP1** ]  ICE  [ **CASP1** ]  specific reversible inhibitor YVAD CHO resulted in a sharp dose and time dependent inhibition of IL 1 beta  [ **IL1B** ]  secretion in the serum.  Ref: 11399517 Eur Cytokine Netw< MedlineTA>Eur Cytokine Netw,
**Neutral relationship**- Interleukin 1 IL 1 beta  [ **IL1B** ]  IL 1beta converting enzyme ICE  [ **CASP1** ]  is a cysteine protease caspase 1  [ **CASP1** ]  that processes inactive 33 kDa pro IL 1beta to the active 17 kDa inflammatory cytokine.  Ref: 11754996 Neurobiol Aging< MedlineTA>Neurobiol Aging,
- caspase 1  [ **CASP1** ]  is the intracellular protease that cleaves the precursors of IL 1 beta  [ **IL1B** ]  and IL 18 into active cytokines.  Ref: 11606779 Proc Natl Acad Sci U S A, 2001
- Amebic lysates have also been shown to activate the precursor to interleukin 1 beta  [ **IL1B** ]  proIL 1beta, mimicking the action of caspase 1  [ **CASP1** ] .  Ref: 12595442 Infect Immun, 2003

**Non-interactive relationship** (e.g. studied together, co-existance, homology, etc.)

- The terminal stages of cell death, including death of neurons, depend on the activation of caspases, specifically caspase 1  [ **CASP1** ]  interleukin 1 beta  [ **IL1B** ]  converting enzyme  [ **CASP1** ] .  Ref: 11544763 Ontogenez< MedlineTA>Ontogenez, 2003
- Cells on oxidized matrix demonstrated enhanced expression of Bax, increased fragmentation of PARP, and diminished apoptosis in the presence of the interleukin 1 beta  [ **IL1B** ]  converting enzyme  [ **CASP1** ]  inhibitor Ac Tyr Val Ala Asp aldehyde.  Ref: 11922616 Biochem Biophys Res Commun, 2002
- Dex inhibited TNFalpha induced messenger ribonucleic acid expression of ICE  [ **CASP1** ] , TNF alpha, and IL 1 beta  [ **IL1B** ]  P <.  Ref: 11397893 J Clin Endocrinol Metab, 2001
- Co expression of PYPAF7 and ASC results in the synergistic activation of caspase 1  [ **CASP1** ]  and a corresponding increase in secretion of interleukin 1 beta  [ **IL1B** ] .  Ref: 12019269 J Biol Chem, 2002
- In the lining like layers and deep stroma of SMLIT, many cells were double positive for active caspase 1  [ **CASP1** ]  and interleukin 1 beta  [ **IL1B** ]  IL 1beta.  Ref: 12111083 Rheumatol Int, 2002
- We sought to determine whether mice deficient in the proinflammatory caspase 1  [ **CASP1** ] , which cleaves precursors of IL 1 beta  [ **IL1B** ]  and IL 18, were protected against ischemic acute renal failure ARF.  Ref: 11342578 J Clin Invest, 2001
- The mRNA expression of IL 18 and IL 1 beta  [ **IL1B** ]  converting enzyme  [ **CASP1** ]  was significantly reduced in unstimulated monocytes from patients with AD P < ;.  Ref: 11590389 J Allergy Clin Immunol, 2001
- Shigella flexneri infection of human macrophages is followed by rapid bacterial escape into the cytosol and secretion of IpaB, which activates caspase 1  [ **CASP1** ]  to mediate cell death and release of mature interleukin IL 1 beta  [ **IL1B** ] .  Ref: 12065527 Infect Immun, 2002
- Using primary cultured murine peritoneal macrophages, we demonstrate that P2X7 receptor activation causes release of IL 1 beta  [ **IL1B** ]  and IL 1 alpha via a common pathway, dependent upon the release of Ca 2 from endoplasmic reticulum stores and caspase 1  [ **CASP1** ]  activity.  Ref: 12626557 J Immunol, 2003
- The LPS induced effects were abrogated by inhibition of caspase 1  [ **CASP1** ] , suggesting that IL 1 beta  [ **IL1B** ]  may mediate the effects of LPS.  Ref: 12609991 J Biol Chem, 2003
- Effect of TNF alpha on the induction of apoptosis in murine macrophages role of interleukin 1 beta  [ **IL1B** ]  converting enzyme  [ **CASP1** ] .  Ref: 12622883 Int J Immunopathol Pharmacol< MedlineTA>Int J Immunopathol Pharmacol, 2003
- Inhibition by SK& ; F 86002 and interleukin 1 beta  [ **IL1B** ]  converting enzyme  [ **CASP1** ]  inhibitor.  Ref: 11399517 Eur Cytokine Netw< MedlineTA>Eur Cytokine Netw, 2003
- These results suggest that lemon pure essential oils may modulate the apoptosis through the activation of the interleukin 1 beta  [ **IL1B** ]  converting enzyme  [ **CASP1** ]  like caspases.  Ref: 12162947 Pharmacol Res, 2002
- As cytoplasmatic adaptor molecules of FAS, e.g. FLIP Fas associated death domain protein FADD like interleukin 1 beta  [ **IL1B** ]  converting enzyme  [ **CASP1** ]  FLICE caspase 8 inhibitory protein, also modulate TRAIL signals, we determined whether chelerythrin affected TRAIL mediated apoptosis.  Ref: 12877678 Br J HaematolBr J Haematol, 2002
- Changes in caspase 3 and caspase 1  [ **CASP1** ] , interleukin 1 beta  [ **IL1B** ]  IL 1 beta  [ **IL1B** ] , and collagen I, III, and IV proteins and mRNA were detected by Western blotting and Northern blotting, respectively.  Ref: 12753292 Kidney Int, 2003
- Intriguingly, unlike the caspase 8 inhibitor cellular FADD like interleukin 1 beta  [ **IL1B** ]  converting enzyme  [ **CASP1** ]  inhibitory protein c FLIP, NDED suppressed TNF mediated apoptosis by inhibiting TNF induced caspase 8 enzymatic activity but not the processing of caspase 8.  Ref: 11346652 J Biol Chem, 2001
- RESULTS Expression of TNF alpha, IL 1 beta  [ **IL1B** ] , caspase 1  [ **CASP1** ]  and NOS2 was found in the cytoplasm and sarcolemma of dystrophic skeletal muscle fibres.  Ref: 12673888 Clin Exp Rheumatol< MedlineTA>Clin Exp Rheumatol, 2001
- Vertex is collaborating with Aventis Pharma AG formerly Hoechst Marion Roussel Inc in the development of pralnacasan, an interleukin IL 1b  [ **IL1B** ]  converting enzyme ICE  [ **CASP1** ]  inhibitor, for the potential treatment of inflammatory diseases 17024 8829 53094.  Ref: 12789619 Idrugs, 2003
- METHODS Immunohistochemical staining of skeletal muscle biopsies 10 DM, 10 SSc, 10 PM, 10 SLE, 10 non inflammatory myopathies for tumour necrosis factor alpha TNF alpha, interleukin 1 beta  [ **IL1B** ]  IL 1 beta  [ **IL1B** ] , activated caspase 1  [ **CASP1** ] , pan macrophage marker CD68, inducible nitric oxide synthase NOS2 and nerve growth factor receptor NGFR.  Ref: 12673888 Clin Exp Rheumatol< MedlineTA>Clin Exp Rheumatol, 2003
- The expression and release of IL 1 beta  [ **IL1B** ] , a postulated downstream effector of ICE  [ **CASP1** ]  mediated apoptosis, were also determined.  Ref: 11397893 J Clin Endocrinol Metab, 2001
- IL 1 beta  [ **IL1B** ]  converting enzyme  [ **CASP1** ]  ICE  [ **CASP1** ] .  Ref: 11606779 Proc Natl Acad Sci U S A, 2001
- We have also assessed the immunoreactivity of interleukin 1 beta  [ **IL1B** ]  converting enzyme  [ **CASP1** ] , caspase 1  [ **CASP1** ] , a key enzyme in the execution of apoptosis, and of P cadherin, which has been suggested as a key adhesion receptor in segregating proliferating keratinocytes.  Ref: 11407986 J Invest Dermatol, 2001
- TNF alpha increased the messenger ribonucleic acid levels of ICE  [ **CASP1** ] , TNF alpha, IL 1 beta  [ **IL1B** ] , bcl 2, and NF kappa B in preadipocytes and adipocytes P <.  Ref: 11397893 J Clin Endocrinol Metab, 2001
- PURPOSE To detect cell specific apoptosis factors, Fas and Fas ligand, and the common intracellular apoptosis modulators, interleukin 1 beta  [ **IL1B** ]  converting enzyme  [ **CASP1** ]  ICE  [ **CASP1** ]  like protease caspase 1  [ **CASP1** ] , Bcl 2, Bcl xL and Bax in lens epithelial cells LEC of human cataracts.  Ref: 11803481 Curr Eye Res, 2001
- CONCLUSION Prominent expression of the proinflammatory factors TNF alpha, IL 1 beta  [ **IL1B** ]  and NOS2 and caspase 1  [ **CASP1** ]  is associated with muscle fibre damage, albeit when expressed to a low degree these factors may, like NGFR, contribute to muscle regeneration and healing.  Ref: 12673888 Clin Exp Rheumatol< MedlineTA>Clin Exp Rheumatol, 2001
- Dual effects of caspase 1  [ **CASP1** ] , interleukin 1 beta  [ **IL1B** ] , tumour necrosis factor alpha and nerve growth factor receptor in inflammatory myopathies.  Ref: 12673888 Clin Exp Rheumatol< MedlineTA>Clin Exp Rheumatol, 2001
- IL 1 beta  [ **IL1B** ]  converting enzyme  [ **CASP1** ]  caspase 1  [ **CASP1** ]  in intestinal inflammation.  Ref: 11606779 Proc Natl Acad Sci U S A, 2001
- Of the 4 cDNA fragments, 2 A1 and D1 did not show sequence homology with the existing genes in the GeneBank, one A2 was 89% homologous with interleukin 1 beta  [ **IL1B** ]  converting enzyme  [ **CASP1** ]  ICE  [ **CASP1** ]  gene, and the other D2 was identical to mouse musculus 45s pre rRNA MM45srRNA gene.  Ref: 11783011 Zhonghua Zhong Liu Za Zhi, 2001
- Using RT multiplex PCR, the expression of the proapoptotic genes interleukin 1 beta  [ **IL1B** ]  IL 1 beta  [ **IL1B** ]  converting enzyme  [ **CASP1** ]  ICE  [ **CASP1** ]  and TNF alpha and the antiapoptotic genes bcl 2, nuclear factor kappa B NF kappa B, and NF kappa B inhibitory subunit, I kappa B, were examined.  Ref: 11397893 J Clin Endocrinol Metab, 2001
- During the early stages of hair follicle development, interleukin 1 beta  [ **IL1B** ]  converting enzyme  [ **CASP1** ]  immunoreactivity was present on all keratinocytes, but virtually disappeared from the proximal hair follicle epithelium later on.  Ref: 11407986 J Invest Dermatol, 2001
- The cellular Fas associated death domain like interleukin 1 beta  [ **IL1B** ]  converting enzyme  [ **CASP1** ]  inhibitory protein long form FLIP L, a structural homologue of caspase 8 lacking caspase activity because of several mutations in the active site, is a potent inhibitor of death receptor induced apoptosis.  Ref: 12215447 J Biol Chem, 2002
